# Stressors and common mental disorder in informal carers – An analysis of the English Adult Psychiatric Morbidity Survey 2007

**DOI:** 10.1016/j.socscimed.2014.09.025

**Published:** 2014-11

**Authors:** Stephen Stansfeld, Melanie Smuk, Juliana Onwumere, Charlotte Clark, Cleo Pike, Sally McManus, Jenny Harris, Paul Bebbington

**Affiliations:** a Centre for Psychiatry, Wolfson Institute of Preventive Medicine, Barts & The London School of Medicine & Dentistry, Queen Mary University of London, Old Anatomy Building, Charterhouse Square, London EC1M 6BQ, UK; b Department of Psychology, King's College London, Institute of Psychiatry, De Crespigny Park, Denmark Hill, London SE5 8AZ UK; c NatCen Social Research, 35 Northampton Square, London EC1V 0AX UK; d Florence Nightingale School of Nursing and Midwifery, Kings College, London, UK; e UCL Mental Health Sciences Unit, 2nd Floor, Charles Bell House 67-73, Riding House Street, London W1W 7EJ, UK

**Keywords:** Carers, Mental health, Stress, Common mental disorders, Social support, Suicide, Socioeconomic status

## Abstract

This study investigates potential explanations of the association between caring and common mental disorder, using the English Adult Psychiatric Morbidity Survey 2007. We examined whether carers are more exposed to other stressors additional to caring – such as domestic violence and debt – and if so whether this explains their elevated rates of mental disorder. We analysed differences between carers and non-carers in common mental disorders (CMD), suicidal thoughts, suicidal attempts, recent stressors, social support, and social participation. We used multivariate models to investigate whether differences between carers and non-carers in identifiable stressors and supports explained the association between caring and CMD, as measured by the revised Clinical Interview Schedule.

The prevalence of CMD (OR = 1.64 95% CI 1.37–1.97), suicidal thoughts in the last week (OR = 2.71 95% CI 1.31–5.62) and fatigue (OR = 1.33 95% CI 1.14–1.54) was increased in carers. However, caring remained independently associated with CMD (OR = 1.58 1.30–1.91) after adjustment for other stressors and social support. Thus caring itself is associated with increased risk of CMD that is not explained by other identified social stressors. Carers should be recognized as being at increased risk of CMD independent of the other life stressors they have to deal with. Interventions aimed at a direct reduction of the stressfulness of caring are indicated. However, carers also reported higher rates of debt problems and domestic violence and perceived social support was slightly lower in carers than in non-carers. So carers are also more likely to experience stressors other than caring and it is likely that they will need support not only aimed at their caring role, but also at other aspects of their lives.

## Highlights

• Caring is associated with an increased risk of depressive and anxiety disorders and suicidal ideation.
• The increased risk of mental-ill-health in carers is not explained by increased life events and low social support.
• Carers are exposed to more debt and domestic violence than the general population.
• Carers should be recognized as being at increased risk of CMD independent of the other life stressors they have to deal with.
• Interventions aimed at a direct reduction of the stressfulness of caring are indicated.

## 1 Introduction

### 1.1 Background

It is estimated that over 6 million people provide informal unpaid care in the UK, a number expected to rise to 9 million by 2037 (Maher and Green, 2002, Carers UK, 2010). Informal caregivers have been described as an ‘invisible healthcare system’ (Arno et al., 1999). Caring for a relative with an illness or disability may result in chronic stress (Schulz and Sherwood, 2008). Research suggests that carers are more vulnerable to both physical (Da Roza Davis and Cowen, 2001, Pinquart and Sorensen, 2007, Perlick et al., 2005) and mental health problems (Gysels et al., 2012, Phillips et al., 2009; Pinquart and Sorensen, 2003, Hirst, 2005), including depression and anxiety (Papastavrou et al., 2012, Cooper et al., 2007, Damjanovic et al., 2007).

### 1.2 Impact of care

The adverse consequences of the caregiver role can be observed across several areas, including disturbances in sleep (Phillips et al., 2009), immunological responses (Damjanovic et al., 2007, Kiecolt-Glaser et al., 1991, Clark et al., 2013), endocrine functioning (Kiecolt-Glaser and Glaser, 1999, Kring et al., 2010), and elevated rates of mortality (Schulz and Beach, 1999). There is evidence to suggest that former carers are also in worse health than non-carers (Lee and Gramotnev, 2007). The negative health sequelae of the caregiving role (e.g. emotional distress) are frequently enduring (Vedhara et al., 2000). The reduction of caregiver burden and improvement in carer wellbeing thus remains a major responsibility for government and public health bodies (Department of Health, 2010, Carers UK, 2010).

### 1.3 Models of caregiving

**Fig. 1:**
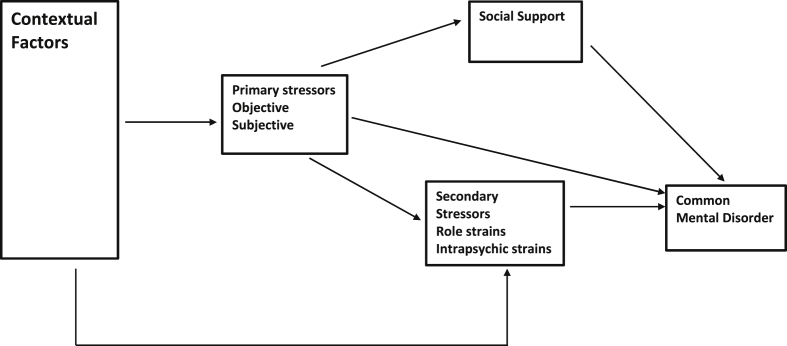
The stress process model.

The stress-process model (Pearlin et al., 1990) and its variants (e.g. Szmukler et al., 1996, Mackay and Pakenham, 2012) have been used to examine the mechanisms whereby caregiving may lead to poor mental health. In this model, factors directly relating to the nature and magnitude of the difficulty of the caring role are labelled as primary stressors (Pearlin et al., 1990, Schulz and Sherwood, 2008) (see Fig. 1, developed following Hilgeman et al. (2009) and Judge et al. (2009)). These can, for example, include dealing with difficult behaviours, cognitive impairment and aggression from the care recipient (Schrag et al., 2006), or with difficult emotions and relational deprivation (Winn et al., 2007, Gilhooly and Whittick, 1989). Caregiving occurs within a social context and background contextual factors such as socioeconomic status, education, gender and ethnicity of the carer can have an important influence on the impact of caregiving on health. The model is also designed to account for the effect of secondary stressors on carer mental health. Secondary stressors include role strains such as strains within relationships with other family members, interference with the carer's occupation, financial strains and restriction of social life due to caring. Intrapsychic strains, such as impacts of caring on dimensions of self-concept such as self-esteem, mastery, role captivity (trapped unwillingly in the carer role), loss of sense of self and competence in caring are also types of secondary stressors. More positively, caring may lead to feelings of personal gain associated with successful achievement of caring which also comes under the heading of secondary stressors. We were unable to measure intrapsychic strains and the detail of strains within family relationships in this study. A significant proportion of mental ill-health in carers may be attributable to stressors associated only indirectly with their caring duties, such as stressful life events, economic strains, job-caregiving conflicts and constriction of social and recreational life (Carers UK, 2007, Carers UK, 2008). For instance, there is evidence that carers often experience other stressors including social and economic disadvantage (Hirst, 2004a), difficulties with finances and employment (Yeandle et al., 2007, Schulz and Martire, 2004, Mangalore and Knapp, 2007) and housing (Gilleard, 1998), and a reduced socioeconomic status (SES) relative to non-caregivers (Pinquart and Sorensen, 2003, Schulz and Sherwood, 2008). The caregiver role often restricts opportunities to engage in social and recreational activities, and this may increase the risk for depression (Neiboer et al., 1998). Thus, caregivers often have smaller social networks and reduced levels of social support (Magliano et al., 2006, McManus et al., 2009), with consequent negative effects on their mental health (Singleton, 2002, Bromley et al., 2004). Thus mental ill-health in carers may be attributable partly to these secondary stressors rather than only the primary stressors involved in aspects of direct caring. The third aspect of the stress process model is access to mediators such as social support. If available, social support can buffer or mediate the strain of the caregiving role and reduce the risk of mental disorder (Pearlin et al., 1990).

### 1.4 Carer studies

Understanding the precise impact of the caregiving role on carer health outcomes should be an important target for service providers. However, methodological problems such as varying definitions of caring, small self-selected samples, the restriction of samples to those caring for people with specific disabilities (e.g. Awad and Voruganti, 2008, Haley et al., 2010), a lack of non-carer comparison groups and adjustment for socioeconomic position, make it difficult to draw firm conclusions about carer health and lifestyle (Pinquart and Sorensen, 2003, Neugaard et al., 2008, Butterworth et al., 2010). While it is certain that mental health problems in carers will reflect a ‘multitude of stressors’ (Hirst, 2004a), few studies have used multivariate analyses to isolate pathways, whereby the contribution of the caregiving role to common mental disorders (CMD) relative to other known risk factors can be determined (Noh and Turner, 1987, Hirst, 2005, Holmbeck, 1997). This quantifies the association of a wide range of secondary stressors, related to caring, to mental ill-health in a nationally representative sample and seeks to understand how much of the association of primary stressors of caring and mental ill-health can be explained by secondary stressors and the effects of social support as a mediator on the association of caring and mental ill-health (see Fig. 1). We know that caregivers have a higher prevalence of CMD than non-carers (Smith et al., 2014). We therefore hypothesized, first, that carers will report more stressors and lower levels of social support than non-carers, and, secondly, that the association between caregiving and CMD will be partially explained by the higher levels of secondary stressors and lower levels of social support in carers.

## 2 Methods

The Adult Psychiatric Morbidity Survey (APMS) 2007 is a nationally representative random probability survey containing questions on mental and physical health, lifestyle and socio-demographic factors. Households in England were randomly selected using the Small Users Postcode Address File, and one eligible person per household was selected using the Kish grid method (Kish, 1965). 13,171 households were visited, and 57% (7461) of the selected participants agreed to take part (70% of those who could be contacted). The APMS interview consisted of a 90 min computer-assisted personal interview conducted by experienced interviewers (McManus et al., 2009).

### 2.1 Measures

#### 2.1.1 Primary stressors

Carers were identified by a question taken from the 2001 England & Wales census (ONS, 2001), asking whether respondents currently “looked after, gave help or support to family, friends, neighbours or others because they have long term physical or mental ill health or disability.”

#### 2.1.2 Secondary stressors

Stressful life events in the last six months were assessed using the List of Threatening Experiences (Brugha et al., 1985). We constructed measures reflecting the experience of any personal life event and any material life event in the last six months. Illness in a close family member was excluded from the list as potentially being a confounder in the association of caring and common mental disorder. Debt was measured by asking respondents if they had been seriously behind paying for bills and loans in the last 12 months. Ten questions adapted from the Domestic Violence and Abuse module of the British Crime Survey (Walby and Allen, 2004) elicited experiences of non-sexual abuse by a current or previous partner in last 12 months. A dichotomous variable of any domestic violence was derived.

#### 2.1.3 Mediators

Perceived social support was assessed from 7 questions taken from the Health and Lifestyle survey (Cox et al., 1987). Respondents were asked if they thought that family or friends were available for support. Scores were summed and categorized with a maximum score (21) indicating good support, 18–20 moderate support, and <18 low support (Brugha et al., 2003). Social participation, included visits to evening classes, leisure centres, or libraries and active involvement in any recreational or social club, attendance at a local or voluntary community group (at least once month; at least once year; not in last year/never).

#### 2.1.4 Common mental disorders and other health outcomes

Common mental disorders (CMD) were assessed using the Revised Clinical Interview Schedule (CIS-R) (Lewis et al., 1992), a standardised clinical psychiatric interview providing six ICD-10 diagnoses, based on symptoms in the past week. A dichotomous measure based on the presence of any CMD, involving all 6 ICD codes, was derived. CIS-R questions were also used to assess suicidal ideation: whether respondents, in the last week or year, or at any other time, had wished they were dead, had thoughts about suicide without intentions to act, or had attempted to take their life.

Alcohol misuse was measured with the AUDIT scale. A score of ≥8 was used to define a drinking problem (Saunders et al., 1993). Questions on drug use were based on questions from the Diagnostic Interview Schedule (Malgady et al., 1992). Drug use was here defined as acknowledged consumption of any of 14 illicit named drugs in the last 12 months (McManus et al., 2009).

A score of 2 or more from the CIS-R fatigue subscale was used to indicate high levels of fatigue (Lewis et al., 1992). Problems with activities of daily living (ADL) (Martin et al., 1988) were assessed by 7 questions asking about difficulties with activities such as bathing, dressing, taking pills, gardening and decorating. Participants were divided into those who had and who did not have problems with any ADL.

#### 2.1.5 Sociodemographic background factors

Sociodemographic details including age, gender, highest education level, equivalised household income, employment status, Registrar General's Social Class (Standard Occupational Classifications ONS 2000), marital status, number of dependent children in household, and housing tenure were also recorded (McManus et al., 2009).

### 2.2 Statistical analysis

Analysis was conducted using the survey design functions in STATA v13. to account for weighting procedures. Weights were applied to represent the structure of the national population and to account for the probability of selection and non-response (McManus et al., 2009).

All analyses were adjusted for age, gender and socio-economic status (including equivalised household income, education, and occupational class). Risk factors and outcomes were largely treated as dichotomous variables in order to examine the magnitude of effect of multiple variables using odds ratios. Analyses were completed on the weighted complete record subset (*N* = 5170) created when all covariates are completely observed from the fully adjusted multivariate logistic regression model.

**Step 1** involved confirming that the predictor of interest (Caring) was significantly related to the outcome (CMD). The associations of caring with CMD, with individual mental disorders and with fatigue and suicidal ideation were examined using logistic regression.

**Step 2** established whether the predictor (caring) was related to the potential mediating factors (stressors and social supports). The associations between caring, stressors and social support were examined using logistic regression models.

**Step 3**. Those stressor and social support variables satisfying step 2 were added to a series of multivariate logistic regression models to test whether or not the relationship between caring and CMD diminished as a result of their inclusion.

**Step 4**. The mediation effect of personal life events, material life events and perceived social support between CMD and caring (adjusting/controlling for the other covariates in model 4) was investigated separately. As the putative mediators and the outcome are binary variables we followed the method in MacKinnon and Dwyer (1993) to find the coefficients of the mediator paths and their corresponding standard errors. These were then included in three mediation tests, the Sobel, Arorian and Goodman Test. The Sobel assumes the path between caring to the mediator and the path between the mediator to CMD are independent. The Arorian does not assume this and adds the covariance term in the denominator, whereas the Goodman test subtracts it to create an unbiased estimate of the variance. We apply all three to examine how robust inferences are to the chosen method. We put more emphasis on the Arorian test for our conclusions as it has been shown to perform better in a Monte Carlo study (MacKinnon et al., 1995). All three tests make the assumption that the distribution of the product of the regression coefficient is normal; however this is unlikely to be true, it is generally asymmetric with high kurtosis. We thus apply an adjustment to the Arorian test as described by MacKinnon et al. (2002) (first variant) and use the table of critical values to assess significances.

## 3 Results

### 3.1 Caring in the general population

Just over a quarter (weighted N 1367) of the population reported caring duties. Women were more likely to be carers than men (OR = 1.20 95% CI 1.04–1.39), and carers were most likely aged between 55 and 64 years (OR = 2.71 95% CI 1.86–3.94: reference group, 16–24 years). People in the lowest income group (<£10,575) (OR = 1.31 95% CI 1.07–1.61) and those in the second lowest income groups (>=10,575 to <16,195) (OR = 1.52 95% CI 1.24–1.86) were significantly more likely to have caring duties than those in the highest income group. People working in personal service occupations were more likely to be carers (OR = 1.46 95% CI 1.10–1.94: reference group, managers and senior officials) (see Table 1).

**Table 1:** Weighted frequency and percentage of carers from APMS 2007 with corresponding odds ratios and 95% Confidence Interval (CI) for responders characteristics (bold values are significant at the 95% level).

Weighted *N* = 5170	Non-carer *n* (%)	Carer *n* (%)	Total *n* (%)	Odds ratio	95% CI
Gender					
Male	1927 (50.7)	630 (46.1)	2557 (49.5)	**1.00**	
Female	1875 (49.3)	738 (54.0)	2613 (50.5)	**1.20**	**(1.04**–**1.39)**
Age					
16–24	296 (7.8)	60 (4.4)	356 (6.9)	1.00	
25–34	794 (20.9)	149 (10.9)	943 (18.2)	0.93	(0.62–1.38)
35–44	906 (23.8)	294 (21.5)	1200 (23.2)	**1.60**	**(1.10**–**2.35)**
45–54	627 (16.5)	325 (23.8)	952 (18.4)	**2.56**	**(1.76**–**3.73)**
55–64	511 (13.4)	280 (20.5)	791 (15.3)	**2.71**	**(1.86**–**3.94)**
65–74	360 (9.5)	185 (13.5)	545 (10.6)	**2.53**	**(1.70**–**3.77)**
75+	308 (8.1)	74 (5.4)	382 (7.4)	1.19	(0.79–1.80)
Household Income (HI)					
HI ≥ £40,384	933 (24.5)	273 (20.0)	1206 (23.3)	1.00	
£24,700 ≤ HI < £40,384	806 (21.2)	300 (21.9)	1106 (21.4)	**1.27**	**(1.03**–**1.57)**
£16,195 ≤ HI < £24,700	787 (20.7)	266 (19.5)	1054 (20.4)	1.16	(0.94–1.43)
£10,575 ≤ HI < £16,195	624 (16.4)	278 (20.3)	901 (17.4)	**1.52**	**(1.24**–**1.86)**
HI < £10,575	653 (17.2)	251 (18.3)	904 (17.5)	**1.31**	**(1.07**–**1.61)**
Education^b^					
Degree	898 (23.6)	293 (21.4)	1190 (23.0)	1.00	
Teaching^c^	278 (7.3)	116 (8.5)	394 (7.6)	1.28	(0.97–1.69)
A level	561 (14.8)	216 (15.8)	777 (15.0)	1.18	(0.93–1.49)
GCSE^d^	1034 (27.2)	376 (27.5)	1410 (27.3)	1.11	(0.91–1.37)
Foreign^e^	126 (3.3)	45 (3.3)	171 (3.3)	1.10	(0.78–1.55)
No qualifications	906 (23.8)	321 (23.5)	1228 (23.8)	1.09	(0.89–1.33)
Occupation^f^					
Managers and senior officials	589 (15.5)	185 (13.5)	774 (15.0)	1.00	
Professional occupations	494 (13.0)	150 (11.0)	644 (12.5)	0.97	(0.74–1.27)
Associate professional and technical	520 (13.7)	212 (15.5)	732 (14.2)	**1.30**	**(1.00**–**1.69)**
Administrative and secretarial	434 (11.4)	180 (13.2)	614 (11.9)	**1.32**	**(1.02**–**1.72)**
Skilled trades occupations	432 (11.4)	137 (10.0)	569 (11.0)	1.01	(0.78–1.32)
Personal services occupations	286 (7.5)	131 (9.6)	417 (8.1)	**1.46**	**(1.10**–**1.94)**
Sales and customer services occupation	267 (7.0)	97 (7.1)	364 (7.0)	1.16	(0.87–1.56)
Process, plant and machine operative	321 (8.5)	110 (8.0)	431 (8.3)	1.09	(0.80–1.49)
Elementary occupations	460 (12.1)	165 (12.0)	624 (12.1)	1.14	(0.89–1.47)
CMD^g^ (any)	530 (13.9)	284 (20.8)	815 (15.8)	**1.64**^a^	**(1.37**–**1.97)**
GAD^h^	142 (3.7)	81 (5.9)	223 (4.3)	**1.57**^a^	**(1.19**–**2.08)**
MADD^i^	307 (8.1)	154 (11.3)	461 (8.9)	**1.45**^a^	**(1.13**–**1.87)**
Panic	32 (0.8)	25 (1.8)	57 (1.1)	**2.38**^a^	**(1.43**–**3.96)**
OCD	31 (0.8)	14 (1.0)	46 (0.9)	1.34^a^	(0.67–2.66)
Depressive episodes	78 (2.0)	35 (2.6)	113 (2.2)	1.19^a^	(0.79–1.78)
Phobia	46 (1.2)	23 (1.7)	70 (1.4)	1.37^a^	(0.74–2.56)

a Odds ratios adjusted for age, gender, standard occupational classifications, equalised household income and education.

b Highest education level.

c Includes higher national diplomas and nursing.

d Includes GCSE equivalent.

e Includes other.

f Standard occupational classifications.

g Common mental disorder.

h Generalised anxiety disorder.

i Mixed anxiety depression disorder.

### 3.2 Associations between caring and CMD

Carers had an increased risk of having a CMD (OR = 1.64 95% CI 1.37–1.97) adjusting for age, gender, standardised occupational classification, equivalised household income and education. After adjustment for age, gender and SES, carers had increased rates of Generalized Anxiety Disorder (OR = 1.57 95% CI 1.19–2.08), Mixed Anxiety and Depressive Disorder (OR = 1.45 95% CI 1.13–1.87) and Panic Disorder (OR = 2.38 95% CI 1.43–3.96) compared with non-carers (Table 1). Carers were twice as likely to report thinking about suicide in the last week (OR = 2.71 95% CI 1.31–5.62) and more likely to report wishing they were dead in the last week (OR = 2.10 95% CI 1.15–3.82).

### 3.3 Associations between caring, stressors and social support

Table 2 describes the odds of caring in relation to household tenure and various individual measures of stressors and social support. More carers reported domestic violence in the last 12 months (OR = 1.57 95% CI 1.14–2.17), and they were more likely to report debts (OR 1.60 95% CI 1.20–2.13) in the last year than non-carers (Table 2).

**Table 2:** Weighted frequency and percentage of carers from APMS 2007 with corresponding adjusted odds ratios and 95% Confidence Interval (CI): adjusted for age, gender, standard occupational classifications, equalised household income and education (bold values are significant at the 95% level).

Weighted *N* = 5170	Non-carer *n* (%)	Carer *n* (%)	Total *n* (%)	Adjusted odds ratio	95% CI
Household tenure					
Own outright^a^	1013 (26.6)	486 (35.5)	1499 (29.0)	1.00	
Buying with a mortgage	1749 (46.0)	560 (41.0)	2309 (44.7)	**0.75**	**(0.63**–**0.90)**
Rent it^b^	1041 (27.4)	322 (23.5)	1362 (26.4)	**0.79**	**(0.65**–**0.94)**
Life events & alcohol					
Personal life events^c^	53 (1.4)	19 (1.4)	72 (1.4)	0.90	(0.51–1.60)
Material life events^c^	54 (1.4)	15 (1.1)	69 (1.3)	0.81	(0.44–1.48)
Domestic violence	197 (5.2)	93 (6.8)	290 (5.6)	**1.57**	**(1.14**–**2.17)**
Debt	309 (8.1)	144 (10.5)	453 (8.8)	**1.60**	**(1.20**–**2.13)**
Alcohol problem	923 (24.3)	327 (23.9)	1250 (24.2)	1.17	(0.99–1.38)
Informal support					
Perceived low social support	298 (7.8)	104 (7.6)	402 (7.8)	1.02	(0.80–1.31)
Marital status					
Married^d^	2643 (69.5)	1008 (73.7)	3651 (70.6)	1.00	
Single^e^	599 (15.7)	165 (12.1)	764 (14.8)	1.03	(0.80–1.31)
Divorced^f^	561 (14.8)	195 (14.2)	756 (14.6)	**0.78**	**(0.65**–**0.92)**
Social participation					
Community group attendance					
Not in last year/never	2981 (78.3)	954 (69.8)	3935 (76.1)	1.00	
Once a month	549 (14.4)	289 (21.1)	837 (16.2)	**1.52**	**(1.29**–**1.79)**
In the last year	273 (7.2)	125 (9.1)	398 (7.7)	**1.36**	**(1.07**–**1.72)**
Club membership	1662 (43.7)	684 (50.0)	2345 (45.4)	**1.23**	**(1.06**–**1.42)**

a Includes ‘live rent free’.

b Includes ‘pay rent and part mortgage (shared)’.

c One or more events.

d Married or cohabiting.

e Single or never married.

f Widowed, divorced or separated.

Carers were less likely to be divorced/separated/widowed (OR = 0.78 95% CI 0.65–0.92) than non-carers. Carers and non-carers did not differ significantly in visits to evening classes, leisure centres, or libraries, but carers were more likely to participate in a voluntary or local community group in the last year (OR = 1.36 95% CI 1.07–1.72) and were more likely to belong to a recreational, social or community club (OR = 1.23 95% CI 1.06–1.42). More carers reported suffering high levels of fatigue (OR = 1.33 95% CI 1.14–1.54) than non-carers. Self-reported general health did not differ significantly between groups.

### 3.4 Multivariate models of caring and CMD

Four multivariate models tested the relationship between caring and CMD after stressors and social support were added to the models (Table 3). Model 1 demonstrated an association after adjustment for basic socio-demographic factors.

**Table 3:** Adjusted odds ratios and 95% confidence Interval (CI) for CMD, adjusted for standard occupational classification (bold values are significant at the 95% level). Model 1 (M1) adjusted for household tenure, Model 2 (M2) adds adjustment for life events and alcohol, Model 3 (M3) adds adjustment for informal support and Model 4 (M4) adds adjustment for social participation.

Weighted *N* = 5170	M1 AOR^a^	95% CI	M2 AOR^a^	95% CI	M3 AOR^a^	95% CI	M4 AOR^a^	95% CI
Carer	**1.62**	**(1.35**–**1.94)**	**1.52**	**(1.26**–**1.83)**	**1.55**	**(1.29**–**1.87)**	**1.58**	**(1.30**–**1.91)**
Female	**1.53**	**(1.24**–**1.88)**	**1.69**	**(1.35**–**2.11)**	**1.69**	**(1.35**–**2.12)**	**1.64**	**(1.31**–**2.07)**
Age^b^								
25–34	1.49	(0.95–2.34)	**1.63**	**(1.03**–**2.58)**	1.56	(0.98–2.48)	1.56	(0.98–2.48)
35–44	1.28	(0.82–2.00)	1.45	(0.92–2.27)	1.35	(0.85–2.13)	1.38	(0.87–2.18)
45–54	**1.58**	**(1.00**–**2.49)**	**2.02**	**(1.27**–**3.20)**	**1.82**	**(1.14**–**2.92)**	**1.88**	**(1.17**–**3.01)**
55–64	0.91	(0.56–1.49)	1.23	(0.75–2.02)	1.10	(0.66–1.84)	1.14	(0.68–1.90)
65–74	**0.54**	**(0.31**–**0.91)**	0.75	(0.44–1.28)	0.63	(0.37–1.10)	0.67	(0.39–1.17)
75+	**0.46**	**(0.27**–**0.78)**	0.69	(0.40–1.18)	**0.52**	**(0.30**–**0.92)**	**0.55**	**(0.31**–**0.96)**
Household Income^c^ (HI)								
£24,700 ≤ HI < £40,384	0.92	(0.69–1.22)	0.89	(0.66–1.20)	0.89	(0.66–1.20)	0.89	(0.66–1.20)
£16,195 ≤ HI < £24,700	1.25	(0.95–1.66)	1.20	(0.90–1.60)	1.16	(0.87–1.54)	1.15	(0.86–1.53)
£10,575 ≤ HI < £16,195	**1.46**	**(1.07**–**2.00)**	1.35	(0.97–1.87)	1.33	(0.96–1.84)	1.30	(0.94–1.80)
HI < £10,575	**1.78**	**(1.30**–**2.43)**	**1.45**	**(1.06**–**2.00)**	1.37	(0.99–1.88)	1.34	(0.98–1.85)
Education^d^								
Teaching^e^	0.98	(0.66–1.45)	0.92	(0.62–1.35)	0.89	(0.61–1.31)	0.89	(0.60–1.31)
A level	1.12	(0.81–1.55)	1.08	(0.77–1.51)	1.08	(0.77–1.51)	1.06	(0.76–1.48)
GCSE^f^	1.16	(0.86–1.55)	1.10	(0.82–1.48)	1.13	(0.84–1.51)	1.09	(0.82–1.46)
Foreign^g^	0.82	(0.49–1.40)	0.86	(0.50–1.48)	0.88	(0.52–1.50)	0.85	(0.49–1.47)
No qualifications	**1.51**	**(1.10**–**2.07)**	**1.47**	**(1.06**–**2.04)**	**1.47**	**(1.06**–**2.02)**	1.37	(0.99–1.90)
Household tenure^h^								
Buying with a mortgage	0.91	(0.70–1.18)	0.89	(0.68–1.16)	0.88	(0.67–1.15)	0.86	(0.66–1.13)
Rent it^i^	**1.64**	**(1.29**–**2.08)**	**1.35**	**(1.05**–**1.73)**	1.26	(0.97–1.62)	1.21	(0.94–1.58)
Life events & alcohol								
Personal life events^j^			**3.83**	**(2.32**–**6.34)**	**3.90**	**(2.30**–**6.59)**	**3.81**	**(2.25**–**6.48)**
Material life events^j^			0.69	(0.34–1.40)	0.67	(0.33–1.38)	0.67	(0.33–1.38)
Domestic violence			**2.85**	**(2.11**–**3.87)**	**2.71**	**(1.99**–**3.69)**	**2.72**	**(1.99**–**3.72)**
Debt			**2.61**	**(2.08**–**3.29)**	**2.46**	**(1.95**–**3.11)**	**2.49**	**(1.97**–**3.15)**
Alcohol problem			**1.38**	**(1.11**–**1.72)**	**1.34**	**(1.08**–**1.68)**	**1.34**	**(1.08**–**1.67)**
Informal support								
Low social support^k^					**2.03**	**(1.55**–**2.66)**	**2.00**	**(1.54**–**2.61)**
Marital status^l^								
Single^m^					1.09	(0.87–1.37)	1.08	(0.86–1.36)
Divorced^n^					**1.57**	**(1.28**–**1.92)**	**1.56**	**(1.27**–**1.92)**
Social participation								
Community group^o^								
Once a month							1.06	(0.82–1.37)
In the last year							0.84	(0.59–1.19)
Club membership							**0.73**	**(0.61**–**0.88)**

a CMD odds ratio adjusted for other covariates in the model and SOC.

b Reference category (RC) 16–24.

c RC: HI_£40,384.

d Highest education level RC: Degree.

e Includes higher national diplomas and nursing.

f Includes GCSE equivalent.

g Includes other.

h RC: Own outright including live rent free.

i Includes pay rent and part mortgage (shared).

j RC: 0 Events, against having one or more events.

k Perceived support RC: High/medium against low.

l RC: Married or cohabiting.

m Single or never married.

n Widowed, divorced or separated.

o Group attendance RC: Not in the last year/never.

In model 2 the addition of personal and material life events, debts, alcohol problems and domestic violence diminished the relationship between caring and CMD, (OR = 1.52 95% CI 1.26–1.83). Domestic violence, a personal life event in the last six months, debt, income less than £10,575 and no educational qualifications were significant independent predictors of CMD.

The addition of informal social support (perceived social support and marital status) in model 3 did not further reduce the association between caring and CMD (OR = 1.55 95% CI 1.29–1.87). Perceived social support was a significant independent risk factor for CMD, with those reporting low support at a 2.03 (95% CI 1.55–2.66) increased odds of CMD. In model 4 where community group participation and club membership were added to the model, there was again no substantial change in the association between caring and CMD (OR = 1.58 95% CI 1.30–1.91). Being a member of a club was associated with a 0.73 (95% CI 0.61–0.88) reduction in odds of CMD. Debt, a personal life event, domestic violence, marital status, and moderate/low perceived support also remained independently associated with CMD in the final model.

In Table 4 we report whether personal life events, material life events or perceived social support mediated the association of caregiving and CMD. There was no evidence to support mediation tested by Sobel, Aroian or Goodman tests. The data did not appear to be sensitive to the choice of mediating test. We applied the MacKinnon et al. (2002) (first variant) method to the Aroian test statistic by using the table to find the proportions under the curve beyond the test value we obtained and doubling it for a two tail test. The method is only approximate as our sample size is larger than the table shows. The method supports our previous findings that personal life events, material life events and perceived social support do not have a significant mediating effect between caring and CMD separately.

**Table 4:** Sobel, Aroian and Goodman test results for model 4, investigating the mediating effect of personal, material life events and perceived social support between being a carer and having a common mental disorder (performed separately), weighted *N* = 5170.

Mediator tested	Test statistic	Standard error	*p*-value
Personal life events			
Sobel test	−0.37	0.01	0.71
Aroian test	−0.36	0.01	0.72
Goodman test	−0.37	0.01	0.71
Material life events			
Sobel test	0.78	0.00	0.43
Aroian test	0.66	0.00	0.51
Goodman test	1.01	0.00	0.31
Perceived low social support			
Sobel test	−0.19	0.00	0.85
Aroian test	−0.19	0.00	0.85
Goodman test	−0.20	0.00	0.85

## 4 Discussion

The current findings confirm that caregiving remains an independent risk factor for CMD in a model including stressors and social support. The odds of CMD were 58% higher in carers than in non-carers. The effect of caring on CMD is comparable to the effects of living in a low income household, or leaving school without qualifications. Carers were also more likely to report difficulties with debt and domestic violence.

In multivariate models, the inclusion of stressors had the largest impact on the relationship between caring and CMD, but only reduced the odds of CMD in carers from 1.62 to 1.52. The addition of social support variables did not reduce the odds of CMD in carers further. Stressors and social support variables included in the models showed independent effects on CMD after adjustment for all other variables.

### 4.1 The stress process

It is likely that some of the stressors reported by carers are a direct result of the caring role. Elevated rates of trauma have been reported in carer populations (Noble and Schenk, 2008). In severe mental health disorders, carers are also more likely to be the targets of aggressive behaviour from care recipients (Nielssen et al., 2007, Nordstrom and Kullgren, 2003). Further, approximately 4% of the current sample acknowledged suicidal thoughts in the preceding year. Suicidal ideation has been reported in other carer populations such as dementia carers, albeit at higher rates (O'Dwyer et al., 2013). However, since details about the source of some of the reported stressors were not given in the current survey, it is difficult to establish whether they were a direct result of caring duties. The caring role is likely to be stressful at least in part because of the accumulation of a variety of small ties and obligations, which would not be captured by our measures. There does appear to be a dose effect: caring has been shown to involve a greater risk of mental disorder in those carrying out heavy caring duties and spending more time caring per week, and carrying out caring at home (Hirst, 2004a). The physiological response associated with recurrent exposure to stressors and the stress proliferation involved in caregiving may lead to allostatic processes attempting to compensate for the effects of stress that increase the risk of physical ill-health and mortality rates in caregivers (McEwen and Seeman, 1999, Pearlin, 2010, Schulz and Beach, 1999) although this could also be mediated through CMD.

The cross-sectional nature of our analyses also constrains inferences about causality. However, whether a consequence of caring or a confounding factor, several stressors are more frequent in people who have taken up a caring role than in non-carers.

### 4.2 Comparison to other studies

The risk of CMD found in carers in this study is comparable to other general population surveys that include a representative sample of carers (Singleton, 2002, Maher and Green, 2002, Hirst, 2004b). Hirst (2004a) also found a similar association between caring and mental health.

Panic and anxiety disorders were most prevalent amongst carers in the current study, as noted by others (Vanderwerker et al., 2005, Cooper et al., 2007). Fatigue has also been widely reported in carers, which may be an indirect reflection of the degree of primary stressors but may also be seen as an intermediate outcome on the pathway to CMD (Singleton, 2002, Maher and Green, 2002).

It is not very surprising that carers reported stressful life events. The finding that carers report more debt problems is consistent with other studies highlighting financial difficulties in caregiver groups (Carers UK, 2007, Carers UK, 2008, Mangalore and Knapp, 2007). Our finding that debt problems might contribute to the relation between caring and CMD is in line with studies linking debt with CMDs (Meltzer et al., 2013), and with theories suggesting vulnerability to CMD in people of lower socioeconomic status (Lorant et al., 2002). It is suggested elsewhere that financial difficulties increase with time caring, possibly partly because caring may interfere with paid work (Arksey and Hirst, 2005).

Reduced levels of perceived support and social participation with a club membership did not account for the higher prevalence of CMD in carers in multivariate models. This is encouraging evidence that carers from this sample were not subject to significant restrictions in their social and recreational activities.

### 4.3 Methodological issues

This study corrects for some previous methodological problems. Bias in sample selection was reduced by using a representative sample, including anyone that defined themselves as a carer. Earlier studies have used comparable definitions, encompassing all carers, but have reported smaller proportions of carers (Horsley et al., 1998; Green and Maher, 2002; Singleton, 2002). As our definition of caring does not seem more inclusive this might reflect a proportionate increase in carers (Smith et al., 2014). Few epidemiological studies of carers have included the range of secondary stressors measured in this study (Butterworth et al., 2010, Pinquart and Sorensen, 2003, Neugaard et al., 2008). However, in this study we did not have all the variables we needed for the stress process model. We did not have several role strain variables such as precise indicators of difficulties in relationships with other family members in relation to caring, and we did not have measurements of intrapsychic strains-this may have weakened our ability to test the full effects of secondary stressors.

Reverse causation may explain some of our findings, e.g. the higher prevalence of CMD in carers may lead to a tendency to report more stressful life events (Kessler, 1997) or lower levels of social support (Sarason et al., 1991). Longitudinal data would assist in the attribution of causation. Our study was limited in that it lacked information on locus of care or type of care and its statistical power was insufficient to investigate subgroups of carers. The stressors and supports for those caregiving to people may differ by disease cared for and by age and relationship (e.g. spouse, child or parent) (Awad and Voruganti, 2008, Papastavrou et al., 2012, Zegwaard et al., 2013). Finally, our study might have missed those with severe mental distress or more time consuming caring duties as they are less likely to take part in a general population survey.

There has been a call for developing comprehensive models of mental disorder in caregiving (Pinquart and Sorensen, 2003, Neugaard et al., 2008). Astrength of our study is the incorporation of an extended range of stressors in a single model. Our analyses were shaped by the stress process model (e.g. Pearlin et al., 1990), and examined possible mediating pathways in caregiver CMD. We mainly focused on stressors indirectly associated with caring (secondary stressors and contextual factors) but our findings leave much of the relationship between caring and CMD to be explained. Primary stressors directly related to the caring relationship are likely to explain more of the caring and CMD relationship, and require to be further investigated alongside indirect factors. Personality characteristics (e.g. Lautenschlager et al., 2013; Hooley and Hiller, 2000) and coping styles (e.g. Papastavrou et al., 2012, Onwumere et al., 2011) of the caregiver would also be useful to include in future surveys (Schrag et al., 2006, Pinquart and Sorensen, 2003). In some studies caregiving is associated with positive benefits to mental health and increased mastery and sense of achievement (Pearlin et al., 1990). We were not able to assess these aspects of caregiving which are important because they emphasise that caregiving is not invariably a negative experience.

## 5 Conclusion

Caring is moderately associated with CMD, an association that is not explained by social support and stressors. All carers in the English population should be recognized as being potentially at increased risk of CMD, although for some caregiving has positive effects on mental health. This study provided evidence that carers are also exposed to high levels of stressors such as debt problems, domestic violence, and stressful life events which are associated with increased risk of CMD in both carers and non-carers; these secondary stressors did not explain the association of caring and CMD. It was not clear in this cross-sectional study whether the stressors examined were caused by caring duties but this study shows that there is inequity between carers and non-carers that may lead to mental health problems. Carers may experience a multitude of risk factors for CMD which have an additive effect. To ensure optimal health outcomes for care recipients, whose functioning and community tenure may be dependent upon the care provided by an informal carer, it would seem important for health professionals to be aware of how additional sources of stress might add to the existing stress of caring. Strategies are needed to assess carer stressors in the wider life roles and the context in which they provide care. Further research that makes explicit the process by which the caregiving role can lead to CMD is likely to aid the development of specialist, evidence based carer interventions that can apply to a broad range of mental and physical conditions.

## References

[bib1] Arksey H., Hirst M. (2005). Unpaid carers' access to and use of primary care services. Prim. Health Care Res. Dev..

[bib2] Arno P.S., Levine C., Memmott M.M. (1999). The economic value of informal caregiving. Health Aff. (Millwood).

[bib3] Awad A.G., Voruganti L.N. (2008). The burden of schizophrenia on caregivers: a review. Pharmacoeconomics.

[bib4] Bromley J., Hare D.J., Davison K., Emerson E. (2004). Mothers supporting children with autistic spectrum disorders: social support, mental health status and satisfaction with services. Autism.

[bib5] Brugha T., Bebbington P., Tennant C., Hurry J. (1985). The list of threatening experiences: a subset of 12 life event categories with considerable long-term contextual threat. Psychol. Med..

[bib6] Brugha T.S., Morgan Z., Bebbington P., Jenkins R., Lewis G., Farrell M., Meltzer H. (2003). Social support networks and type of neurotic symptom among adults in British households. Psychol. Med..

[bib7] Butterworth P., Pymont C., Rodgers B., Windsor T.D., Anstey K.J. (2010). Factors that explain the poorer mental health of caregivers: results from a community survey of older Australians. Aust. N. Z. J. Psychiatry.

[bib8] Carers UK (2007).

[bib9] Carers UK (2008).

[bib10] Carers UK (2010).

[bib11] Clark M.C., Nicholas J.M., Wassira L.N., Gutierrez A.P. (2013). Psychosocial and biological indicators of depression in the caregiving population. Biol. Res. Nurs..

[bib12] Cooper C., Balamurali T.B.S., Livingston G. (2007). A systematic review of the prevalence and covariates of anxiety in caregivers of people with dementia. Int. Psychogeriatr..

[bib13] Cox B.D., Blaxter M., Buckle A.L.J., Fenner N.P., Golding J.F., Gore M., Huppert F.A., Nickson J., Roth M., Stark A., Wadsworth M.E.J., Wichelow M. (1987).

[bib14] Da Roza Davis J.M., Cowen P.J. (2001). Biochemical stress of caring. Psychol. Med..

[bib15] Damjanovic A.K., Yang Y., Glaser R., Kiecolt-Glaser J.K., Nguyen H., Laskowski B., Zou Y., Beversdorf D.Q., Weng N.P. (2007). Accelerated telomere erosion is associated with a declining immune function of caregivers of Alzheimer's disease patients. J. Immunol..

[bib16] Department of Health (2010).

[bib17] Gilhooly M.L., Whittick J.E. (1989). Expressed emotion in caregivers of the dementing elderly. Br. J. Med. Psychol..

[bib18] Gilleard C. (1998).

[bib19] Gysels M., Evans N., Menaca A., Andrew E., Toscani F., Finetti S., Pasman H.R., Higginson I., Harding R., Pool R. (2012). Culture and end of life care: a scoping exercise in seven European countries. PLoS One.

[bib20] Haley W.E., Roth D.L., Howard G., Safford M.M. (2010). Caregiving strain and estimated risk for stroke and coronary heart disease among spouse caregivers: differential effects by race and sex. Stroke.

[bib21] Hilgeman M., Durkin D.W., Sun F., DeCoster J., Allen R.S., Gallagher-Thompson D., Burgio L.D. (2009). Testing a theoretical model of the stress process in Alzheimer' s caregivers with race as a moderator. Gerontologist.

[bib22] Hirst M. (2004).

[bib23] Hirst M. (2004).

[bib24] Hirst M. (2005). Carer distress: a prospective, population-based study. Soc. Sci. Med..

[bib25] Holmbeck G.N. (1997). Toward terminological, conceptual, and statistical clarity in the study of mediators and moderators: examples from the child-clinical and pediatric psychology literatures. J. Consult Clin. Psychol..

[bib26] Hooley J.M., Hiller J.B. (2000). Personality and expressed emotion. J. Abnorm. Psychol..

[bib27] Horsley S., Barrow S., Gent N., Astbury J. (1998). Informal care and psychiatric morbidity. J. Public Health Med..

[bib28] Judge K.S., Menne H.L., Whitlatch C.J. (2009). Stress process model for individuals with dementia. Gerontologist.

[bib29] Kessler R.C. (1997). The effects of stressful life events on depression. Annu. Rev. Psychol..

[bib82] Kiecolt-Glaser J.K., Glaser R. (1999). Chronic stress and mortality among older adults. JAMA.

[bib30] Kiecolt-Glaser J.K., Dura J.R., Speicher C.E., Trask O.J., Glaser R. (1991). Spousal caregivers of dementia victims: longitudinal changes in immunity and health. Psychosom. Med..

[bib31] Kish L. (1965).

[bib32] Kring S.I., Brummett B.H., Barefoot J., Garrett M.E., Ashley-Koch A.E., Boyle S.H., Siegler I.C., Sorensen T.I., Williams R.B. (2010). Impact of psychological stress on the associations between apolipoprotein E variants and metabolic traits: findings in an American sample of caregivers and controls. Psychosom. Med..

[bib33] Lautenschlager N.T., Kurz A.F., Loi S., Cramer B. (2013). Personality of mental health caregivers. Curr. Opin. Psychiatry.

[bib34] Lee C., Gramotnev H. (2007). Life transitions and mental health in a national cohort of young Australian women. Dev. Psychol..

[bib35] Lewis G., Pelosi A.J., Araya R., Dunn G. (1992). Measuring psychiatric disorder in the community: a standardized assessment for use by lay interviewers. Psychol. Med..

[bib36] Lorant V., Deliege D., Eaton W., Robert A., Philippot P., Ansseau M. (2002). Socioeconomic inequalities in depression: a meta-analysis. Am. J. Epidemiol..

[bib37] Mackay C., Pakenham K.I. (2012). A stress and coping model of adjustment to caring for an adult with mental illness. Commun. Ment. Health J..

[bib38] MacKinnon D.P., Dwyer J.H. (1993). Estimating mediated effects in prevention studies. Eval. Rev..

[bib39] MacKinnon D.P., Warsi G., Dwyer J.H. (1995). A simulation study of mediated effect measures. Multivar. Behav. Res..

[bib40] MacKinnon D.P., Lockwood C.M., Hoffman J.M., West S.G., Sheets V. (2002). A comparison of methods to test mediation and other intervening variable effects. Psychol. Methods.

[bib41] Magliano L., Fiorillo A., Malangone C., De Rosa C., Maj M., National Mental Health Project Working Group (2006 Mar). Social network in long-term diseases: a comparative study in relatives of persons with schizophrenia and physical illnesses versus a sample from the general population. Soc. Sci. Med..

[bib42] Maher J., Green H. (2002).

[bib43] Malgady R.G., Rogler L.H., Tryon W.W. (1992). Issues of validity in the diagnostic interview schedule. J. Psychiatr. Res..

[bib44] Mangalore R., Knapp M. (2007). Cost of schizophrenia in England. J. Ment. Health Policy Econ..

[bib45] Martin J., Meltzer H., Elliot D. (1988).

[bib47] McEwen B.H., Seeman T.E., Adler N.E., Marmot M., McEwen B.H. (1999). Socioeconomic Status and Health in Industrialized Nations: Social Psychological and Biological Pathways.

[bib48] McManus S., Meltzer H., Brugha T., Bebbington P., Jenkins R. (2009).

[bib49] Meltzer H., Bebbington P., Brugha T., Farrell M., Jenkins R. (2013). The relationship between personal debt and specific common mental disorders. Eur. J. Public Health.

[bib50] Neugaard B., Andresen E., McKune S.L., Jamoon E.W. (2008). Health related quality of life in an a national sample of caregivers: findings from the behavioural risk factor surveillance system. J. Happiness Stud..

[bib51] Nieboer A.P., Schulz R., Matthews K.A., Scheier M.F., Ormel J., Lindenberg S.M. (1998). Spousal caregivers' activity restriction and depression: a model for changes over time. Soc. Sci. Med..

[bib52] Nielssen O.B., Westmore B.D., Large M.M., Hayes R.A. (2007). Homicide during psychotic illness in New South Wales between 1993 and 2002. Med. J. Aust..

[bib53] Noble A.J., Schenk T. (2008). Posttraumatic stress disorder in the family and friends of patients who have suffered spontaneous subarachnoid hemorrhage. J. Neurosurg..

[bib54] Noh S., Turner R.J. (1987). Living with psychiatric patients: implications for the mental health of family members. Soc. Sci. Med..

[bib55] Nordstrom A., Kullgren G. (2003). Victim relations and victim gender in violent crimes committed by offenders with schizophrenia. Soc. Psychiatry Psychiatr. Epidemiol..

[bib56] O'Dwyer S.T., Moyle W., Zimmer-Gembeck M., De L.D. (2013). Suicidal ideation in family carers of people with dementia: a pilot study. Int. J. Geriatr. Psychiatry.

[bib57] Office for National Statistics (2000).

[bib58] Office for National Statistics (2001).

[bib59] Onwumere J., Kuipers E., Bebbington P., Dunn G., Freeman D., Fowler D., Garety P. (2011). Coping styles in carers of people with recent and long-term psychosis. J. Nerv. Ment. Dis..

[bib60] Papastavrou E., Charalambous A., Tsangari H., Karayiannis G. (2012). The burdensome and depressive experience of caring: what cancer, schizophrenia, and Alzheimer's disease caregivers have in common. Cancer Nurs..

[bib61] Pearlin L.I., Mullan J.T., Semple S.J., Skaff M.M. (1990). Caregiving and the stress process: an overview of concepts and their measures. Gerontologist.

[bib62] Pearlin L.I. (2010). The life course and the stress process: some conceptual comparisons. J. Gerontol. Soc. Sci..

[bib63] Perlick D.A., Hohenstein J.M., Clarkin J.F., Kaczynski R., Rosenheck R.A. (2005). Use of mental health and primary care services by caregivers of patients with bipolar disorder: a preliminary study. Bipolar Disord..

[bib64] Phillips A.C., Gallagher S., Hunt K., Der G., Carroll D. (2009). Symptoms of depression in non-routine caregivers: the role of caregiver strain and burden. Br. J. Clin. Psychol..

[bib65] Pinquart M., Sorensen S. (2003). Differences between caregivers and noncaregivers in psychological health and physical health: a meta-analysis. Psychol. Aging.

[bib66] Pinquart M., Sorensen S. (2007). Correlates of physical health of informal caregivers: a meta-analysis. J. Gerontol. B Psychol. Sci. Soc. Sci..

[bib67] Sarason B., Pierce G.R., Shearin E.N., Sarason I.G., Waltz J.A., Poppe L. (1991). Perceived social support and working models of self and actual others. J. Personal. Soc. Psychol..

[bib68] Saunders J.B., Aasland O.G., Babor T.F., de la Fuente J.R., Grant M. (1993 Jun). Development of the Alcohol Use disorders Identification test (AUDIT): WHO collaborative project on early detection of persons with harmful alcohol consumption–II. Addiction.

[bib69] Schrag A., Hovris A., Morley D., Quinn N., Jahanshahi M. (2006). Caregiver-burden in parkinson's disease is closely associated with psychiatric symptoms, falls, and disability. Parkinsonism Relat. Disord..

[bib70] Schulz R., Beach S.R. (1999). Caregiving as a risk factor for mortality: the caregiver health effects study. JAMA.

[bib71] Schulz R., Martire L.M. (2004). Family caregiving of persons with dementia: prevalence, health effects, and support strategies. Am. J. Geriatr. Psychiatry.

[bib72] Schulz R., Sherwood P.R. (2008 Sep). Physical and mental health effects of family caregiving. Am. J. Nurs..

[bib73] Singleton N. (2002).

[bib74] Smith L., Onwumere J., Craig T., McManus S., Bebbington P., Kuipers E. (2014). Mental and physical illness in caregivers: results from an English national survey sample 2007. Br. J. Psychiatry.

[bib75] Szmukler G.I., Herrman H., Colusa S., Benson A., Bloch S. (1996). A controlled trial of a counselling intervention for caregivers of relatives with schizophrenia. Soc. Psychiatry Psychiatr. Epidemiol..

[bib76] Vanderwerker L.C., Laff R.E., Kadan-Lottick N.S., McColl S., Prigerson H.G. (2005). Psychiatric disorders and mental health service use among caregivers of advanced cancer patients. J. Clin. Oncol..

[bib77] Vedhara K., Shanks N., Anderson S., Lightman S. (2000). The role of stressors and psychosocial variables in the stress process: a study of chronic caregiver stress. Psychosom. Med..

[bib78] Walby S., Allen J. (2004).

[bib79] Winn S., Perkins S., Walwyn R., Schmidt U., Eisler I., Treasure J., Berelowitz M., Dodge L., Frost S., Jenkins M., Johnson-Sabine E., Keville S., Murphy R., Robinson P., Yi I. (2007). Predictors of mental health problems and negative caregiving experiences in carers of adolescents with bulimia nervosa. Int. J. Eat Disord..

[bib80] Yeandle S., Bennett C., Buckner L., Fry G., Price C. (2007).

[bib81] Zegwaard M.I., Aartsen M.J., Grypdonck M.H., Cuijpers P. (2013). Differences in impact of long term caregiving for mentally ill older adults on the daily life of informal caregivers: a qualitative study. BMC Psychiatry.

